# Novel Vaccine Adjuvants

**DOI:** 10.1155/2013/835105

**Published:** 2013-10-22

**Authors:** Anshu Agrawal, Mohammad Owais, Udai P. Singh

**Affiliations:** ^1^Division of Basic and Clinical Immunology, Department of Medicine, University of California, Irvine, CA 92697, USA; ^2^Interdisciplinary Biotechnology Unit, Aligarh Muslim University, Aligarh, Uttar Pradesh 202002, India; ^3^Department of Pathology, Microbiology and Immunology, University of South Carolina, School of Medicine, Building 1, Room B 42, 6439 Garners Ferry Road, Columbia, SC 29208, USA

Vaccines still remain the most successful method for protection and eradication against diseases. However, to avoid harmful effects associated with whole organism vaccines, new vaccine candidates are composed of parts of an organism, and therefore these are weakly immunogenic. Adjuvants are essential components of vaccines that nonspecifically stimulate the immune system, particularly the innate immune system cells, to enhance the immunogenicity of vaccines. Initially the major function of adjuvants such as alum was to allow sustained presence of antigens by preventing their degradation *in vivo*. Recent advances have, however, demonstrated that success of alum as an adjuvant is also due to its ability to activate the innate immune system cells. Significant progress in the last decade has increased our understanding of the innate immune system which is highly complex and can be activated via a wide array of receptors to generate different immune responses. The nature of adaptive immune response, quality, and quantity are governed by how the innate immune responses are activated. Novel adjuvants are therefore targeted to receptors expressed on antigen-presenting cells (APCs) such as dendritic cells (DCs) to activate the innate immune system. DCs express an array of pathogen recognition receptors (PRRs) so that they can recognize any threat to the body. Examples of PRRs include the TLRs, CLRs, and NLRs. Apart from preventing infections, vaccinations are also being used as therapy against tumors. Targeting antigens to APCs along with adjuvants allows the induction of immune response against tumors. The manuscript by L. Arribillaga et al., in this tissue, demonstrates that fusion of an antigen to the extra domain A from fibronectin (EDA) targets antigens to TLR4-expressing cells such as DCs leading to their activation. The approach is also effective for cross-presentation as well as the induction of antiviral/tumor immunity. Furthermore, the approach is universal as conjugation of EDA to streptavidin allows any biotinylated antigens to be used as immunogen for vaccination purposes. In contrast to the approach above, N. Kojima et al. use oligomannose-coated liposomes to target and stimulate APCs. The oligomannose on the liposomes targets antigens to the C-type lectin receptor (CLR) and is effective in inducing CTLs and/or Th1 cells. This approach is also universal because both lipophilic and hydrophilic antigens can be incorporated in the liposomes. Contrary to these surface receptor targeting methodologies, C. A. Colaco et al. discuss the merits of using heat shock proteins (HSPs) as vaccine adjuvants because HSPs are not just stimulators of innate immunity but can also traffic antigens into APCs, facilitating the induction of specific acquired immune responses. 

Since the new generations of adjuvants being developed are highly specific in the type of CD4T, CD8T, or B-cell response they can stimulate, therefore, recent approaches for vaccine development are focused on utilizing a combination of adjuvants to activate the various components of immune system to generate an effective memory response. The manuscript by E. Mata et al. provides an excellent example of a combinatorial approach in which they discuss that a successful malaria vaccine may require a combination of adjuvants which can activate different parts of the immune system. The review also provides an excellent overview of various types and properties of adjuvants in both preclinical and clinical use with particular focus on malaria vaccines. The manuscript by M. J. McCluskie et al. further highlights that a combination effect is better at providing protection. These authors demonstrate that a combination of CpG composed of synthetic oligodeoxynucleotides (ODN) containing immunostimulatory CpG motifs (CpG) and ISCOMATRIX adjuvant (ISCOMATRIX), composed of saponin, phospholipid, and cholesterol which possess both immunostimulatory and delivery properties is more effective than either one alone in inducing responses to different antigens including ova, hemagglutinin (HA), and hepatitis B antigen (HBSag). 

Recent advances suggest that nutritional supplements can also stimulate the immune system and therefore can be used as adjuvants. A. K. Radhakrishnan et al. compared the adjuvant effect of different forms of vitamin E on tetanus toxoid responses. Their observations suggest that plant derived vitamin E such as the tocotrienols are more effective immune modulators than *α*-tocopherols at enhancing the production of cytokines that promote cell-mediated (TH1) immune response. Lastly, development of *in vivo* models is required because it can be used to determine the efficacy of adjuvants in stimulating the helper, cytotoxic, and B-cell memory responses. The manuscript by S. Tufail et al. discusses the merits of using *Listeria* infection as model for studying CD8 T-cell memory. Apart from highlighting the various immunological components required to generate effective CD8 T-cell memory, the authors also provide a glimpse into few adjuvants which have been found to be effective in this model.

*Anshu Agrawal* *Mohammad Owais* *Udai P. Singh*

